# Editorial: Nutrients, Neurotransmitters and Brain Energetics

**DOI:** 10.3389/fnins.2020.568937

**Published:** 2020-11-17

**Authors:** Adriana Ximenes-da-Silva, Rubem Carlos Araújo Guedes

**Affiliations:** ^1^Instituto de Ciências Biológicas e da Saúde, Universidade Federal de Alagoas, Maceió, Brazil; ^2^Departamento de Nutrição, Universidade Federal de Pernambuco, Recife, Brazil

**Keywords:** nutrition, brain energetics, neuroprotection, neuroinflammation, brain excitability, essential fatty acid (EFA), nutrients, neurotransimitter

Deficiency or overabundance of nutrients has a marked effect on brain functionality. Lasting effects are observed even when a nutritional insult occurs early in life, i.e., during brain development (Morgane et al., 1978; Guedes, 2011). Nutrient deficits, especially related to protein-energy undernutrition, have been extensively studied, but the specific effects of diet supplementation on brain functionality are growing in interest, particularly supplementation with lipids and their derivatives. Lifestyle and eating habits have changed worldwide over the last few decades. The consumption of high-palatable diets that are rich in carbohydrates and fats in association with a sedentary lifestyle has dramatically increased, and therefore, metabolic syndrome and neurodegenerative diseases are reaching epidemic proportions (Berthoud, 2012; O'Neil et al., 2014; Afshin et al., 2017).

The number of studies investigating the beneficial and deleterious effects of nutrients, especially fatty acids, on brain functions has increased, and dietary fatty acids are critical for brain excitability, metabolism, and behavior. From dietary manipulations of proteins, carbohydrates and lipids in *in vitro* studies of brain-cultured cells and clinical studies, the articles presented in this special issue investigate changes of brain excitability, metabolism, brain fatty acid membrane composition, and cognitive functions. The *in vivo* and *in vitro* studies that are presented below support the importance of lipids, or their derivatives, and proteins in normal brain excitability, development, cognition maturation and improvement and examine the role of novel food sources or nutraceutical compounds as emerging strategies to improve brain functionality at specific stages of the lifespan.

de Melo et al. studied diet manipulation with different fatty acid compositions in rats receiving diets supplemented with avocado (*Persea americana* Mill.) pulp and oil. Compared to the non-supplemented control group, maternal diet supplementation during gestation and lactation with avocado pulp and oil induced changes in the fatty acid composition of brain cell membranes, with higher total poly-unsaturated fatty acids (PUFA) in the brains of rat adult offspring. Avocado pulp supplementation resulted in a higher docosahexaenoic acid (DHA) proportion in the hippocampus. Oil- and pulp-supplemented diets anticipated in the offspring the appearance of reflex responses, and avocado pulp supplementation amplified six of seven tested reflexes. In another study, Queiroz et al. demonstrated memory improvement in rat offspring from pregnant and lactating dams that were treated with conjugated linoleic acid (CLA). Reflex maturation anticipation and reduced locomotor activity were also present in rats whose mothers received 3% CLA diet supplementation.

Mendoza et al. and Lerner et al. studied the effects of fatty acids on brain excitability, metabolism and cell degeneration using a combination of cotinine plus krill oil administered via gavage or palmitoylethanolamide intraperitoneal injection, respectively. Krill oil is a source of PUFAs that is extracted from small marine crustaceans and exhibits higher DHA bioavailability than fish oil, and it has beneficial health effects on hyperlipidemia, inflammation, and cognitive function in rodents and humans (Xie et al., 2019). Cotinine is the main metabolite of nicotine, and it was used as a therapeutic agent to reduce neuroinflammation, enhance memory and reduce the effects of neurodegenerative diseases, such as Parkinson's disease (Barreto et al., 2015). C57BL/6J mice were subjected to restraint stress and treated for 21 consecutive days with a mixture of cotinine and krill oil. Treated animals exhibited a significant reduction in depressive-like behavior and cognitive impairment. An increased number of GFAP-positive cells was demonstrated in the hippocampal dentate gyrus of stressed mice treated with cotinine and krill oil. These results suggest that the association of cotinine and krill oil improves cognitive impairment and cell damage in the hippocampus of stressed animals and prevents depressive-like behavior and brain cells degeneration (Mendoza el al.).

Nutraceutical use of fatty acids and its derivatives in neuroprotection is increasing. Lerner et al. studied sub-chronic, prophylactic palmitoylethanolamide (PEA), which is a fatty acid amide present in a variety of food sources, such as egg yolks, soybeans, and peanuts, in C57BL/6J mice. Tissue lipidomic and transcriptomic analyses demonstrated that intraperitoneal administration of PEA differentially regulated neuroinflammatory pathways in the spleen and hippocampus. There was a downregulation of splenic pro-inflammatory signaling cytokines and a decrease in hippocampal neuroinflammatory pathways. A transcriptional downregulation of brain-derived neurotrophic factor (BDNF) and upregulation of Crh genes, which modulate hippocampal excitability, were also observed. These observations collectively represent new insights into the molecular changes of the prophylactic use of PEA to decrease the pro-inflammatory environment and modulate brain excitability, which suggests beneficial actions of this nutraceutical in neuroprotection, neuroinflammation, and convulsive disorders.

Behavioral and metabolic changes are also present during menopause due to ovarian hormone variability, which lead to a decreased metabolic rate and mood disorders (Gordon et al., 2015). Dornellas et al. studied the effects of high-fat diets enriched in lard or fish-oil on metabolism, behavior and the monoaminergic system were studied in ovariectomized rats. A lard (saturated fatty acid-rich) diet was more obesogenic than a fish-oil diet and had effects on the hippocampal monoaminergic system. A reduced expression of 5-HT_1B_ mRNA and increased expression of 5-HT_2C_ receptors was observed after treatment with a high-fat, fish oil-based diet. The serotonin-mediated hypothalamic hypophagia response was restored in ovariectomized rats receiving a high-fat fish-oil diet. Both diets reduced anxiety, but the lard diet induced a depressive-like behavior. Dietary long-chain omega-3 fatty acid consumption and behavioral changes in humans is an active research topic. Some evidence, as showed in this issue by Darcey et al., indicates a possible role of omega-3 fatty acids intake in modulating impulse control behavior in adolescents. Based on a Food Frequency Questionnaire and Go/No-Go functional magnetic resonance imaging (MRI) study, the authors evidenced an inverse relationship between the energy-adjusted omega-3 Index and activity in the dorsal cingulate cortex, which suggests that the intake of omega-3 fatty acids in adolescents is associated with a better ability to manage impulse control.

Two review papers in this issue examined diet-related nutritional imbalance and neuropsychiatric disorders: Meira et al. and Melo et al.. Melo et al. discussed the main role of dietary lipid-mediated tissue inflammation on cognitive deficits and behavioral changes. A cross-talk between peripheral proinflammatory cytokines, such as IL-6, IL-1β, and TNFα and activation of brain inflammation is suggested as part of the potential mechanisms involved in cognitive deficits due to an increased intake of diets rich in saturated fatty acids associated with a reduced intake of PUFAs. A very high omega-6/omega-3 ratio promotes brain inflammation, activation of microglia, and reduces tryptophan availability to produce serotonin, which may contribute to depressive-like symptoms. Further evidence demonstrated that omega-3 PUFAs prevented microglial activation and neuroinflammation and maintained normal synaptic function. Accordingly, Hu et al. demonstrated the neuroprotective effects of propionate, a short chain fatty acid precursor in lipid biosynthesis, in a study of neurite outgrowth in the SH-SY5Y neuroblastoma cell line treated with haloperidol. Neuropeptide Y mediated the neuroprotective effect of propionate on neurite outgrowth because treatment with NPY-siRNA (siNPY) suppressed the haloperidol-induced impaired neurite length, which indicates that propionate plays a role in neuroprotection.

Multiple pathways are involved in neuroinflammation/neuroprotection. For example, constant exposure to infrasound, which are inaudible to human ears (frequencies below 20 Hz), results in neuroinflammation and hearing damage. The role of astrocyte activation in mediating neuroinflammation was evaluated in rats and cultured astrocytes exposed to 16 Hz, 150 dB infrasound (Shi et al.). The results showed the participation of the fibroblast growth factor 2/fibroblast growth factor receptor 1 (FGF2/FGFR1) pathway, which inhibited astrocyte activation and reduced the levels of pro-inflammatory cytokines *in vitro* and *in vivo*. Neuroinflammation is also a characteristic of the epileptic brain. One of the most common treatments to manage refractory epilepsy is the use of ketogenic diets (KDs). A variety of long- and medium-chain fatty acids are used in KDs in the search for more appropriate combinations of anaplerotic fatty acids. Meira et al. present the classical mechanisms related to metabolic and neurotransmitter changes under KD, and new perspectives on the role of gut microbiota on the anti-seizure and anti-inflammatory effects of KD are mentioned. In their review article Poff et al. discuss new evidences of ketone bodies alone acting on anti-seizure properties of KD. Furthermore, Granado-Rojas et al. showed in neuronal cells of rat dentate gyrus an increased K+/Cl- cotransporter (KCC2) expression which could be involved in the well-known GABA-mediated inhibition of brain excitability.

The consumption of protein-restricted diets during intrauterine and early postnatal life profoundly affects brain development and function. Current research presented in this issue in various papers shows the long-term effects of maternal protein-restricted diets in the first (F1) and second (F2) generations of rats. Mitochondrial function and neurotransmitter shifts were associated with anxiety-related behaviors and attentional deficits in adulthood. Mokler et al. showed that *in vivo* microdialysis of neurotransmitters to F1 restricted-protein rats revealed interhemispheric differences in the concentration of monoamines, with lower norepinephrine (NE) and dopamine (DA) levels in the right than left ventromedial prefrontal cortex (vmPFC) of malnourished rats. Increased levels of serotonin (5-HT) in the left vmPFC were also observed. In another investigation published in this issue, Newman et al. demonstrated attentional deficits in prenatal protein-malnourished rats, which were less distracted than well-nourished rats when confronted with predictable and unpredictable visual distractors. These results suggest more cognitive rigidity in malnourished rats. The noradrenergic system is associated with this cognitive rigidity because animals with selective noradrenergic deafferentation of the prelimbic cortex show the same behavioral change. Protein-restricted diets affect brain regions differently when malnutrition occurs during F1 or F2 rat generations, which was demonstrated in this issue by Santana et al.. A maternal low-protein diet affected brainstem mitochondrial bioenergetics in young male F1 rats, with the F1 progeny showing increased reactive species production and decreased antioxidant capacity. The antioxidant capacity was upregulated in adulthood in F2 generation rats. These data demonstrate an attempt of F2 generation rats to recover from the nutritional insult of the F1 generation. In this issue, Vega et al. using electroencephalographic data from children exposed to protein energy malnutrition (PEM) in the first year of life proposed a new classification procedure to evaluate the effects of early malnutrition on brain electrical activity.

Environmental factors tightly influence nutritional status and cognitive brain functions. Mendes et al. studied the association of soft (powder) diet, age, and environment impoverishment in young and aged mice. The reduction of masticatory activity due to intake of the powder diet changed mouse exploration patterns in the open field test, and environmental impoverishment contributed to the modification of exploratory patterns of locomotor activity, which declined with age.

The creation of new perspectives for improving nutritional status is represented in this issue by the search for safe food sources and supplements, as reviewed by Sinha et al.. These authors reviewed the nutritional properties of Spirulina and reinforced its use as a food source against protein malnutrition. Spirulina is a blue green alga that has been used as food source since the ancient times of the Aztecs and Mayans. Recent studies confirmed that Spirulina was an important source of protein (60 to 70%), essential fatty acids, vitamins, and minerals. Sinha et al. reviewed its effects and molecular mechanisms against oxidative stress, protein malnutrition, and neuroinflammation and showed beneficial effects of a Spirulina-supplemented diet in neuroprotection. Notably, the effects of plant derivatives, or synthetic anxiolytic/psychostimulant compounds, on behavioral disorders are also discussed in this issue. Stress, depression-like and anxiety-like behaviors are associated with imbalanced neurotransmitter systems and related cognitive deficits. The consumption of GABA-containing tea (2.01 mg/200 mL cup) had a positive effect in young people with self-reported stress and reduced heart rate variability (Hinton et al.). An experimental study demonstrated that matured hop bitter acids orally administered for 6 consecutive days improved depression-like behavior in mice and suppressed hippocampal neuroinflammation. Vagus nerve stimulation may mediate the effects of hop bitter acids and lead to increases in hippocampal norepinephrine levels via the *locus coeruleus* (Fukuda et al.). Zinc compounds also presented an antidepressant and anxiolytic effect in diabetic rats suggesting zinc supplementation as potential beneficial compound to improve neurobehavioral deficits in diabetic animals as demonstrated by Cavalcanti et al..

Francisco and Guedes demonstrated a cholinergic system action on behavioral parameters and brain electrical activity using chronic (21-day) pilocarpine administration in rats. The sub-convulsing dose (45 mg/kg) increased anxiety-like behavior and reduced the velocity of the propagation of cortical spreading depression (CSD), which is a pathophysiological phenomenon related to brain excitability disorders. Animals grown in unfavorable lactation conditions (suckling in a large litter size, 15 pups/dam) exhibited a more marked pilocarpine decelerating effect on CSD propagation compared to normal-sized litters (9 pups/dam), which suggests a differential change in brain excitability according to nutritional status. The same research group (Francisco et al.) demonstrated that these pilocarpine-effects also occurred at an older age. The authors demonstrated changes in blood glucose levels and the immunoreactivity for glial fibrillary acidic protein (GFAP)-containing astrocytes, and ionized calcium-binding adapter molecule 1 (Iba1)-containing microglia. Finally, these authors presented data suggesting that treatment with taurine attenuated the pilocarpine effects.

Changes in brain excitability were also reported in humans who were chronic alcohol consumers. This condition modified visual acuity in humans and led to deficits in spatial luminance contrast sensitivity and color vision. Visual impairment was not reversed after long-term alcohol abstinence, as demonstrated by Martins et al.. Furthermore, Lacerda et al. studying Amazonian population environmentally exposed to mercury contamination proved visual dysfunction mainly characterized by lower visual perimetric area in this population. Additional evidence suggests that ascorbate levels in retinal neuronal cells are fundamental to glutamatergic neurotransmission. The *in vitro* study in chick retinal cells (Portugal et al.) suggested a role of dopamine regulation of ascorbate release, which requires glutamate release and activation of α-amino-3-hydroxy-5-methyl-4-isoxazolepropionic acid (AMPA)/kainate receptors. Malnutrition and vitamin C deficiency are recurrent in chronic alcoholics, and mechanistic hypotheses, as presented by Portugal et al. are needed to better understand the role of the bioavailability of ascorbate to neuronal cells in the retina and visual function.

Astrocytes play a central role in neuroprotection, brain metabolism, cellular communication, water transportation, regulation of neurotransmitters, ion concentrations, and brain diseases. Glucose destination in neural cells depends on cell metabolism, glucose supply and brain activity. Lactate produced and released by astrocytes during increased brain activity binds to monocarboxylate transporters (MCTs) or hydroxy-carboxylic acid receptor 1 (HCAR1) on post-synaptic neurons and astrocytic perivascular processes regulating synaptic communication and plasticity (Gonçalves et al.). Neuroplasticity mediated via brain-derived neurotrophic factor (BDNF) production during high-intensity interval training, which is characterized by exercise in relatively short bursts of vigorous activity, are also discussed in this issue by Jiménez-Maldonado et al..

Metabolic and vascular changes occur during the development of stroke and brain tumors, such as glioblastoma, which is the most aggressive CNS cancer. Regulation of the astrocytic expression of aquaporin 4 (AQP4) mediates the mechanisms of neuroprotection. Costa et al. used *in vivo* and *in vitro* approaches and demonstrated modulation of AQP4 expression via the thyroid hormone triiodothyronine (T3). This effect was observed during CNS development with T3 exerting a biphasic effect. AQP4 expression was reduced in postnatal early life, and its expression increased in the brains of young mice. T3 treatment to the GBM-95 glioblastoma cell line reduced AQP4 expression, which suggests that T3 reduction of AQP4 in astrocytes would prevent brain damage related to water flux changes in cerebral parenchyma. Data from rodent studies also showed improvement of brain functionality after stroke, which is a long-term disability related to brain hemodynamic and water transport changes, when animals were treated with *Panax ginseng* Meyer (Araliaceae). Neuroprotective effects were partially related to the action of ginseng on AQP4 reduced expression (Liu et al.). Therefore, astrocytic modulation of AQP4 expression seems to participate in the mechanisms implicated in the attenuation of brain damage related to stroke and glioblastoma cancer.

The articles presented in this special issue focus on dietary-related, metabolism-associated and neurotransmitter-dependent effects on brain excitability and behavioral changes, especially related to protein and fatty acid diet composition. The ingestion of a polyunsaturated omega-3-enriched diet showed beneficial effects, including improving metabolism, monoaminergic neurotransmitter system and depression-like and anxiety-like behaviors, and nutrient-deficient diets or high-fat saturated diets increased neuroinflammation and promoted attentional deficits, anxiety depressive-like behavior and cognitive rigidity. Regarding interventions based on the administration of nutritional compounds or drugs, additional studies demonstrating beneficial effects of the dose-response phenomenon known as hormesis, improving health and enhancing lifespan as a function of the adaptive plasticity of the organisms (Brunetti et al., 2020; Di Rosa et al., 2020), are surely wanted. We conclude that the interesting conjunction of the studies presented in this Research Topic provide a nice discussion about the underlying mechanisms of the neuroprotection/neuroinflammation relationship.

## Author Contributions

AX-d-S conceived of the presented idea. AX-d-S and RG wrote the manuscript. Both authors contributed to the article and approved the submitted version.

## Conflict of Interest

The authors declare that the research was conducted in the absence of any commercial or financial relationships that could be construed as a potential conflict of interest.
